# Nanostructured Polymeric Materials with Protein-Repellent and Anti-Caries Properties for Dental Applications

**DOI:** 10.3390/nano8060393

**Published:** 2018-06-01

**Authors:** Ning Zhang, Ke Zhang, Xianju Xie, Zixiang Dai, Zeqing Zhao, Satoshi Imazato, Yousif A. Al-Dulaijan, Faisal D. Al-Qarni, Michael D. Weir, Mark A. Reynolds, Yuxing Bai, Lin Wang, Hockin H. K. Xu

**Affiliations:** 1 Department of Orthodontics, School of Stomatology, Capital Medical University, Beijing 100050, China; dentistzhang112@163.com (N.Z.); tuzizhangke@163.com (K.Z.); dentist.x@163.com (X.X.); 13552873721@163.com (Z.D.); 18710097336@163.com (Z.Z.); 2 Department of Advanced Oral Sciences and Therapeutics, University of Maryland School of Dentistry, Baltimore, MD 21201, USA; yaldulaijan@umaryland.edu (Y.A.A.-D.); FAlqarni1@umaryland.edu (F.D.A.-Q.); michael.weir@umaryland.edu (M.D.W.); MReynolds@umaryland.edu (M.A.R.); 3 Department of Biomaterials Science, Osaka University Graduate School of Dentistry, Osaka 565-0871, Japan; imazato@dent.osaka-u.ac.jp; 4 Department of Substitutive Dental Sciences, College of Dentistry, Imam Abdulrahman bin Faisal University, Dammam 31441, Saudi Arabia; 5 VIP Integrated Department, Stomatological Hospital of Jilin University, Changchun 130021, China; 6 Center for Stem Cell Biology & Regenerative Medicine, University of Maryland School of Medicine, Baltimore, MD 21201, USA; 7 Marlene and Stewart Greenebaum Cancer Center, University of Maryland School of Medicine, Baltimore, MD 21201, USA; 8 Department of Mechanical Engineering, University of Maryland Baltimore County, Baltimore County, MD 21250, USA

**Keywords:** dental restorations, polymer nanocomposites, protein-repellent, anti-biofilm, tooth mineral regeneration, caries inhibition

## Abstract

Dental caries is prevalent worldwide. Tooth cavity restorations cost more than $46 billion annually in the United States alone. The current generation of esthetic polymeric restorations have unsatisfactory failure rates. Replacing the failed restorations accounts for 50–70% of all the restorations. This article reviewed developments in producing a new generation of bioactive and therapeutic restorations. This includes: Protein-repellent and anti-caries polymeric dental composites, especially the use of 2-methacryloyloxyethyl phosphorylcholine (MPC) and dimethylaminododecyl methacrylate (DMAHDM); protein-repellent adhesives to greatly reduce biofilm acids; bioactive cements to inhibit tooth lesions; combining protein-repellency with antibacterial nanoparticles of silver; tooth surface coatings containing calcium phosphate nanoparticles for remineralization; therapeutic restorations to suppress periodontal pathogens; and long-term durability of bioactive and therapeutic dental polymers. MPC was chosen due to its strong ability to repel proteins. DMAHDM was selected because it had the most potent antibacterial activity when compared to a series of antibacterial monomers. The new generation of materials possessed potent antibacterial functions against cariogenic and periodontal pathogens, and reduced biofilm colony-forming units by up to 4 logs, provided calcium phosphate ions for remineralization and strengthening of tooth structures, and raised biofilm pH from a cariogenic pH 4.5 to a safe pH 6.5. The new materials achieved a long-term durability that was significantly beyond current commercial control materials. This new generation of bioactive and nanostructured polymers is promising for wide applications to provide therapeutic healing effects and greater longevity for dental restorations.

## 1. Introduction

Dental caries is the most prevalent human infectious disease worldwide [1,2,3,4]. In the United States alone, nearly 200 million tooth cavity restorations are performed each year, costing more than $46 billion annually [5]. Approximately 50–70% of all restorations are performed to replace the failed restorations [1,2,6]. Furthermore, with people living longer and keeping more of their teeth, the need for tooth restorations is increasing rapidly [7]. Elderly people often have gingival recession, tooth root exposures, and reduced saliva flow, leading to root caries [8]. Polymeric composites are popular because of their tooth-colored esthetics, ability for direct placement, and photopolymerization [4,9]. The performance of dental polymeric composites has been greatly improved in the past decades [10,11,12,13,14,15]. However, composite restorations tend to accumulate more oral biofilms and plaques that lead to caries [16,17]. Oral biofilms produce acids, which, coupled with microgaps at the tooth-restoration interfaces, can cause secondary caries and restoration failures [4,6,18,19].

Therefore, researchers have been developing novel antibacterial dental polymers containing quaternary ammonium methacrylates (QAMs) to inhibit bacterial growth and plaque formation [20,21,22,23]. In the mouth in vivo, a clean polymer surface is quickly coated with salivary pellicles that contain salivary proteins [24]. This protein coating is the prerequisite for oral bacteria attachment to the surface [25]. The attachment of early colonizers, such as mutans streptococcus to salivary pellicles, represents the first step in biofilm formation. Therefore, making the polymer surface protein-repellent would reduce or eliminate biofilm formation. Following this line of thinking, Müller et al. immobilized a protein-repellent material, poly(ethylene glycol) (PEG) and two pyridinium group-containing methacrylate monomers, to silicon wafer surfaces, which indeed, had much less adsorbed proteins [26]. Other studies investigated 2-methacryloyloxyethyl phosphorylcholine (MPC), which is a methacrylate with a phospholipid polar group in the side chain [27]. MPC has strong protein-repellency, and has been incorporated into artificial blood vessels, hip joints, and microfluidic devices [28,29,30,31]. Several MPC-containing medical devices have won the approvals of the United States Food and Drug Administration, and have been used clinically [28,31]. Recently, protein-repellent dental composites, bonding agents, cements, and coatings were developed, for the first time, to repel bacterial adhesion, decrease acid production, and protect tooth structures [32]. This article reviews the new generation of nanostructured, bioactive, and therapeutic dental polymeric materials with protein-repellent and anti-caries properties.

## 2. Protein-Repellent and Anti-Caries Polymeric Dental Composites

To suppress oral biofilm/plaque buildup and increase the restoration’s longevity, novel QAMs were developed and incorporated into dental polymers [20,21,33,34]. Imazato et al. invented 12-methacryloyloxydodecylpyridinium bromide (MDPB), which was copolymerized in dental polymers to achieve strong antibacterial activities [21,35,36,37]. In addition, methacryloxylethylcetyl dimethyl ammonium chloride (DMAE-CB), polyethylenimine nanoparticles, and several other novel compositions were also synthesized [34,38,39,40]. However, a primary drawback of polymers containing QAMs is that salivary proteins on the polymer surface would reduce the “contact-killing” efficacy by minimizing direct contacts between bacteria and the polymer surface [20,21,38,41]. Furthermore, salivary proteins on the polymer surface would provide anchor sites for bacterial adhesion, thus increasing biofilm growth and acid production [25]. Therefore, it would be highly desirable to develop a new polymeric composite that can repel proteins and diminish bacterial adhesion.

**Figure 1:**
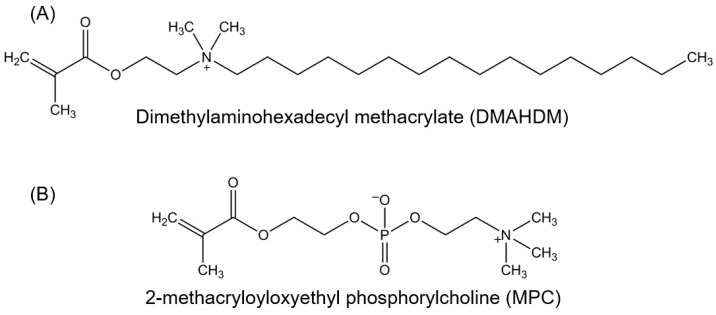
Chemical structures of bioactive monomers. (**A**) Antibacterial monomer dimethylaminohexadecyl methacrylate (DMAHDM), synthesized via a modified Menschutkin reaction; (**B**) Protein-repellent monomer 2-methacryloyloxyethyl phosphorylcholine (MPC).

**Figure 2:**
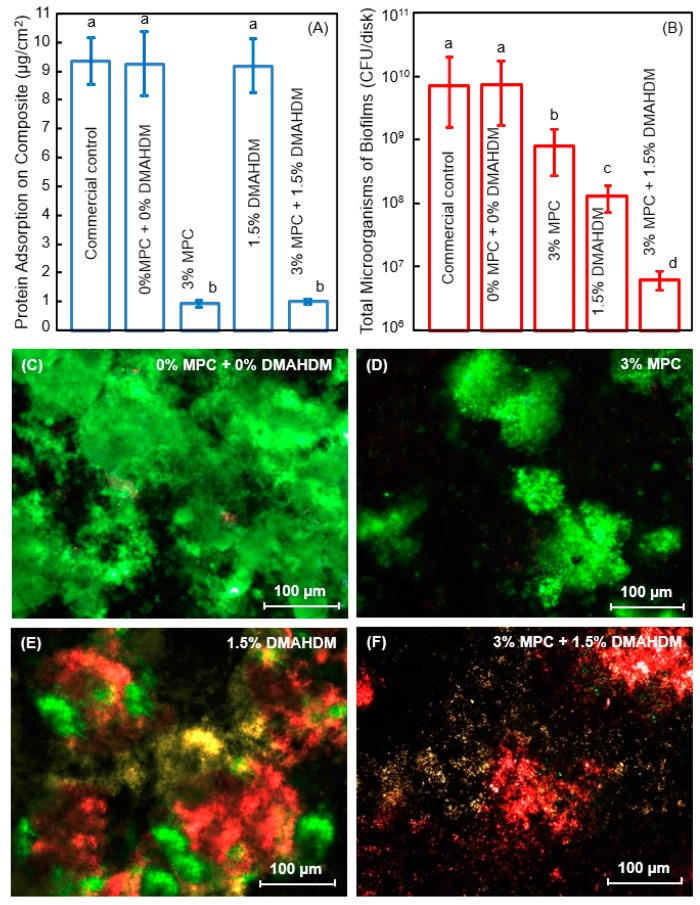
Protein-repellent and antibacterial polymeric composite. (**A**) Protein adsorption onto composites; (**B**) Dental plaque microcosm biofilm cfu of total microorganisms on composites cultured for 2 days. Note the log scale in y axis; (**C**–**F**) Representative live/dead images of biofilms on control composite, and composites with 3% MPC, 1.5% DMAHDM, and 3% MPC + 1.5% DMAHDM. The live bacteria were stained green, and the dead bacteria were stained red. Live and dead bacteria in close proximity yielded yellow/orange colors. In each plot, dissimilar letters indicate values that are significantly different from each other (*p* < 0.05). (Reproduced with permission from [43]. Elsevier, 2015)

Protein-repellency was achieved by incorporating MPC into a dental polymer containing bisphenol glycidyl dimethacrylate (BisGMA) and triethylene glycol dimethacrylate (TEGDMA) [34,35,36,37,38,39,40,41,42]. A new antibacterial monomer dimethylaminododecyl methacrylate (DMAHDM) was synthesized and incorporated into the resin, along with reinforcement glass fillers, to form a composite [43]. Figure 1 shows the chemical structures of DMAHDM and MPC; they both can be copolymerized and covalently bonded with other methacrylate monomers [42,43]. Zhang et al. incorporated 3% by mass of MPC into the composite, which reduced protein adsorption by about an order of magnitude, compared to that with 0% MPC and that of a commercial control composite (Figure 2A) [43]. The flexural strength of the composite containing 3% MPC and 1.5% DMAHDM was 77 ± 5 MPa, similar to 81 ± 5 MPa of a commercial composite without antibacterial and protein-repellent functions (*p* > 0.1) [43]. The composite containing 3% MPC and 1.5% DMAHDM had an elastic modulus of 5.8 ± 0.9 GPa, similar to 6.0 ± 0.7 GPa of the commercial composite (*p* > 0.1) [43].

Using human saliva as inoculum, dental plaque microcosm biofilms were grown on the polymer composites for two days to form a relatively mature biofilm. Zhang et al. measured the colony-forming units (cfu) of total microorganisms, total streptococci, and mutans streptococci on composites containing MPC and DMAHDM [43]. The contained use of MPC and DMAHDM reduced the biofilm cfu by 3 orders of magnitude (Figure 2B). This can be visualized in Figure 2C–F. There were much less, but living, bacteria via MPC (green staining) which reduced bacteria attachment. There were substantial amounts of compromised bacteria (red staining) via DMAHDM, which killed the bacteria via contact-inhibition.

Regarding the mechanism of protein-repellency, MPC contains phospholipid polar groups in the side chain, and phospholipids are a type of lipid in cell membranes [44]. Phospholipid molecules contain hydrophilic heads and hydrophobic tails [44]. Once submerged in water, the phospholipids can orient themselves into a bilayer in which the non-polar tails face the inner area of the bilayer, and the polar heads face outward and interact with the water. Therefore, the MPC polymers are hydrophilic [27]. Hydrophilic surface coatings with MPC incorporation can effectively decrease protein adsorption and bacterial adhesion [28,30,31]. This is because there is an abundance of free water but no bound water in the hydrated MPC polymer. While the presence of bound water would cause protein adsorption, the large amounts of free water around the phosphorylcholine groups contribute to detaching the proteins and reducing their adsorption [45,46].

The combined use of MPC with DMAHDM resulted in stronger reduction in biofilm cfu and more effective killing than each agent alone (Figure 2); this indicated a synergistic effect between MPC and DMAHDM. This effect is related to the mode of antibacterial action of the DMAHDM-containing composite: contact-inhibition [20,21]. It was suggested that when the negatively-charged bacterial cells contact the positively-charged sites of a QAM, the electric balance of the cell membrane could be disturbed, and the bacterium could explode under its own osmotic pressure [38,41,47]. This contact-killing mechanism would indicate that, when a salivary protein pellicle separates the antibacterial polymer surface from the overlaying biofilm, the antibacterial efficacy of the polymer would be reduced [38,41,47]. This was confirmed in several studies showing that a saliva-derived protein coating on the cationic antibacterial surface weakened the bactericidal function [26,48,49]. This is where the advantage of the MPC comes in. MPC can decrease the protein adsorption by an order of magnitude; this would enhance the antibacterial potency of DMAHDM by exposing the polymer surfaces with antibacterial function to kill the bacteria. In return, DMAHDM greatly reduces biofilm buildup (DMAHDM reduced biofilm cfu by 2 logs, Figure 2B) on the polymer surface, thus helping to expose more MPC to repel the incoming proteins. Therefore, the synergy lies in their interactions in that MPC makes DMAHDM more effective, and DMAHDM makes MPC more effective. Their combined use may be beneficial not only to dental polymers, but also to other biomedical materials and tissue engineering scaffolds where the protein-repellent and antimicrobial combination could be highly beneficial to inhibit biofilm growth and prevent infection in the wound site.

## 3. Protein-Repellent Adhesive Resin to Suppress Biofilm Acids

Polymeric composites are filled into tooth cavities and bonded to tooth structures via bonding agents. However, oral biofilms at the tooth-restoration margins can produce acids and cause secondary caries. Indeed, Spencer et al. indicated that the polymer-tooth bonded margin is the “weakest link” of the restoration, and the primary region associated with restoration failures [18]. Therefore, rendering the adhesive polymer protein-repellent would be beneficial to minimize biofilm growth at the margins, thereby to strengthen this “weakest link”. Recently, Zhang et al. developed a protein-repellent bonding agent incorporating MPC for the first time [32,50]. In one example, MPC was incorporated into a commercial bonding system, Scotchbond Multi-Purpose (SBMP). The addition of MPC into SBMP primer and adhesive did not negatively impact the dentin bond strength, while reducing the protein adsorption onto the resin to 1/20 that of a commercial control. This, in turn, substantially decreased the oral bacterial adhesion and biofilm growth on the adhesive resin [50].

**Figure 3:**
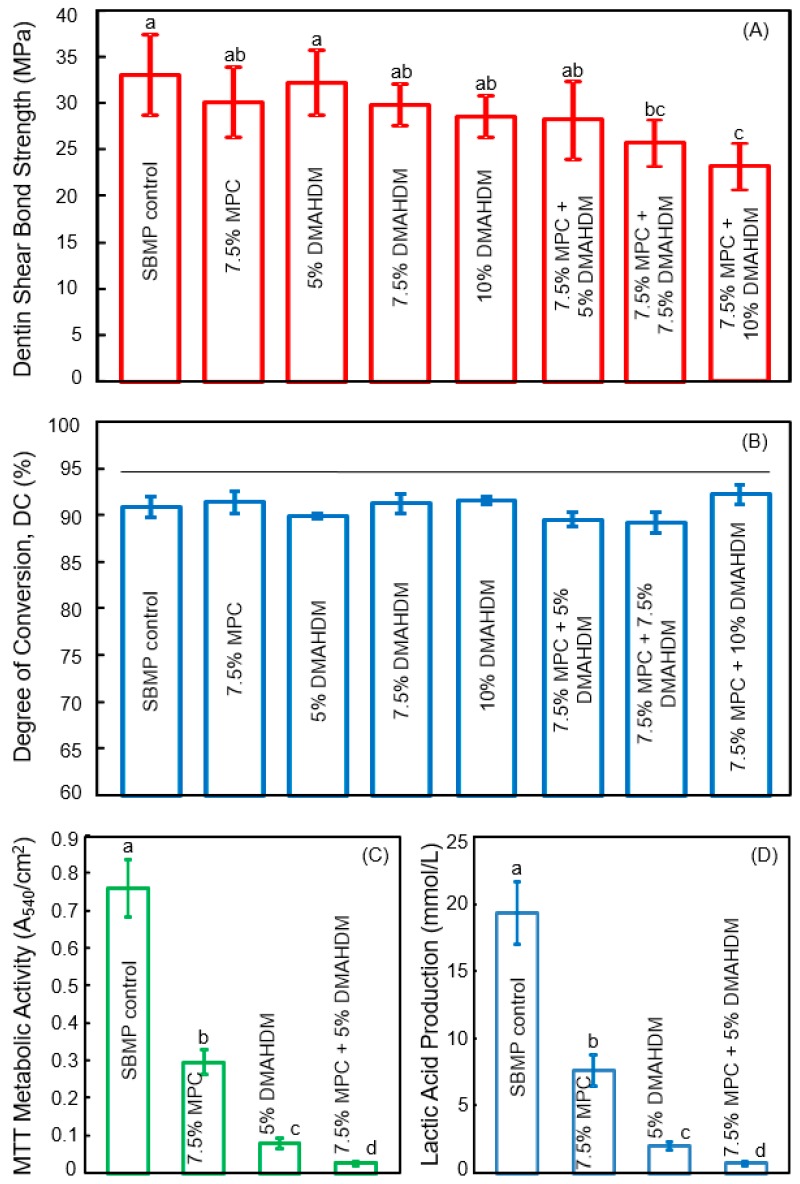
Protein-repellent and antibacterial bonding agent. (**A**) Dentin bond strength; (**B**) Degree of polymerization conversion (DC). The horizontal line indicates values that are not significantly different (*p* > 0.1); (**C**) Metabolic activity of 2-day biofilms; (**D**) Lactic acid production of 2-day biofilms. Values with dissimilar letters indicate significantly different values (*p* < 0.05). (Reproduced with permission from [51]. Elsevier, 2015)

Next, Zhang et al. combined MPC and DMAHDM into the bonding agent to further decrease the biofilm amounts and acid production in the tooth-restoration margins. The MPC mass fraction incorporated into SBMP primer was MPC/(SBMP primer + MPC) = 7.5%. This was selected to produce the strongest protein-repellency, while not compromising the dentin bond strength [50]. Similarly, 7.5% MPC was incorporated into the SBMP adhesive. Then, DMAHDM was incorporated into the SBMP-MPC primer, at DMAHDM/(primer + DMAHDM) of 5%, 7.5%, and 10%, to determine the optimal DMAHDM content when it was combined with 7.5% MPC [51]. Similarly, DMAHDM was added to the SBMP-MPC adhesive at these three concentrations. Since such additions could potentially degrade the bonding agent, the first step was to investigate the effects of MPC + DMAHDM incorporation into the bonding agent on dentin bond strength, and the degree of polymerization conversion. The dentin shear bond results are shown in Figure 3A [51]. Incorporation of up to 7.5% MPC + 5% DMAHDM into both the primer and the adhesive did not adversely affect the dentin bond strength, compared to SBMP control. The degree of conversion is shown in Figure 3B, indicating that the incorporation of MPC and DMAHDM into SBMP did not impact the degree of polymerization conversion.

The second step determined the synergistic effects of MPC + DMAHMD in the bonding agent on biofilm reduction. The results by Zhang et al. are shown in Figure 3C,D [51]. The biofilms on SBMP control had the strongest metabolic activity and produced the most lactic acid among these groups. Incorporation of MPC or DMAHDM, each alone, substantially lowered the metabolic activity and lactic acid of the biofilms. More dramatically, biofilms on the polymer containing 7.5% MPC + 5% DMAHDM had the lowest metabolic activity and the least lactic acid [51].

Beyth et al. suggested that the quaternary amine charge density on the polymer surface is important [38,41]. This is because when the negatively-charged bacteria contact the positively-charged QAM resin, the electric balance of the cell membrane could be disturbed, leading to bacterial destruction [38,41]. Indeed, Murata et al. performed an investigation on antimicrobial polymeric brushes; they showed that high density cationic surfaces killed the bacteria, and long cationic chains could penetrate the bacteria to damage the cell membrane [47]. Li et al. showed that the antibacterial potency of QAMs increased when the alkyl chain length was increased from 5 to 16 [52]. DMAHDM with a chain length of 16 exhibited the most potent antibacterial function among all the tested groups [52]. Li et al. further demonstrated that increasing the quaternary amine charge density on the adhesive polymer surface substantially increased the antibacterial activity [53]. These antibacterial features mean that the synergistic effect of MPC and DMAHDM would be important in the adhesive. The biofilm cfu on the polymer with 7.5% MPC or 5% DMAHDM alone was one or two orders of magnitude lower than that of SBMP control. However, when MPC and DMAHDM were both used, the biofilm cfu was reduced by more than 4 logs, compared to SBMP control [51]. This was likely because MPC could repel proteins, thereby exposing the quaternary amine charge density on the adhesive polymer surface to the bacteria. This could enable the DMAHDM to kill the bacteria and inhibit biofilm growth on the adhesive resin in the restoration marginal region, which is where secondary caries often leads to restoration failure. Therefore, the synergistic enhancement in antibacterial efficacy by the double agents (protein-repellant MPC + antibacterial DMAHDM) was demonstrated not only in composites, but also in bonding agents. Further studies are needed to investigate the protection of the marginal area of the tooth-restorations using the new bonding agent containing both MPC and DMAHDM in an in vivo model. 

## 4. Bioactive Orthodontic Cements That Can Inhibit Tooth Enamel Lesions

Another area where the MPC + DMAHDM method could bring significant benefits is the orthodontic field. The popularity for orthodontic therapy is increasing as more and more people, especially children and teenagers, pursue esthetics and beauty [54]. However, the placement of fixed orthodontic appliances makes oral hygiene more difficult, which leads to the accumulation of biofilm plaque [55]. This can lead to changes in the oral environment, such as more accumulation of microorganisms, biofilm growth, and local acidic pH [56,57]. This could lead to significantly elevated levels of *Streptococcus mutans* (*S. mutans*) and *Lactobacilli* in the mouth [56,57]. Indeed, Enaia et al. showed that the acidic biofilm pH on enamel surfaces adjacent to the fixed appliances could cause demineralization, leading to white spot lesions (WSL) around the orthodontic appliances [58]. Although efforts were made to prevent WSL, 50–70% of patients with fixed orthodontic appliances still had WSL [59,60].

To combat the prevalent occurrence of WSL, oral hygiene and fluoride regimens were recommended [58]. However, these recommendations rely on patient compliance, which is not reliable, especially in children and teenagers [59,60]. Another approach involved the use of resin-modified glass ionomer cements (RMGIs) as the orthodontic cements to bond the bracket to enamel, due to the fluoride-releasing ability and clinically-acceptable bond strength of RMGIs [61]. However, the use of RMGIs as orthodontic cements produced mixed outcomes. For example, Lim et al. suggested that RMGIs remaining around the brackets could have rough surfaces to encourage bacterial attachment [56]. Indeed, their study indicated that there was significantly more *S. mutans* attachments to RMGIs than to resin composites [56]. Furthermore, the orthodontic bracket-enamel junctions around the bracket base often contained gaps of around 10 μm wide, where bacteria could be harbored and biofilms could grow [62]. Therefore, previous studies indicated that RMGIs had little efficacy in preventing demineralization, because the low-pH environment hindered the remineralization, and RMGIs were unable to neutralize acids and increase the local pH [63,64]. 

**Figure 4:**
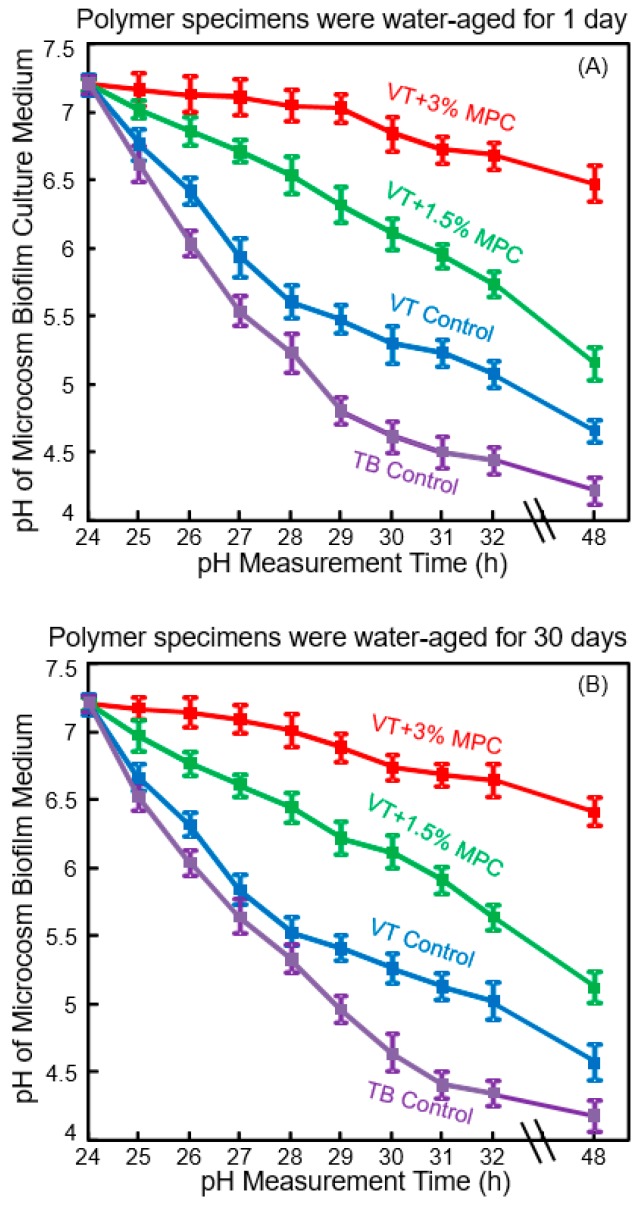
Protein-repellent and antibacterial orthodontic cement. Effect of MPC incorporation on the decrease in pH of culture medium with dental plaque microcosm biofilms: (**A**) pH of medium with biofilms on orthodontic cement disks after being water-aged for 1 day, and (**B**) pH of culture medium with biofilms cultured on the orthodontic cement disks after being water-aged for 30 days. (Reproduced with permission from [66]. Wiley, 2016)

The initial bacterial attachment around the brackets constitutes an important step in WSL formation [65]. The next step is bacterial growth and biofilm formation, producing organic acids to cause WSL [59]. Since the initial salivary protein coating is a prerequisite for bacterial attachment orally [24,25], it would be beneficial to develop novel protein-repellent RMGIs. They could inhibit protein adsorption, diminish bacterial adhesion at the bracket-enamel junctions, and prevent or minimize WSL. Zhang et al. recently reported a novel protein-repellent and fluoride-releasing orthodontic cement by incorporating MPC into a commercial RMGI, Vitremer (referred to as VT) [66]. Another commercial orthodontic cement, Transbond (referred to as TB), served as a non-fluoride-releasing control. The orthodontic cement specimens were water-aged for 1 day or 30 days, and then the microcosm biofilms were grown using human saliva as inoculum, and cultured for two days to form mature biofilms. Figure 4 shows the pH of the biofilm culture medium: (A) 1 day, and (B) 30 days [66]. The pH showed a decreasing trend with culture time due to the biofilms producing acids. However, at 48 h, VT + 3% MPC had a pH that remained at about 6.5. By contrast, for all other groups, the pH decreased with time, reaching 4.7 for VT control, and 4.2 for TB control. Furthermore, even after water-aging for 30 days, similar trends and similar pH values of 2-day biofilms were obtained. This demonstrated that VT + 3% MPC retained its ability to repel bacteria and reduce acid production, and this ability did not decrease from 1 day to 30 days. Therefore, the novel protein-repellent method reduced protein adsorption on VT, thereby substantially reducing oral biofilm formation and lactic acid production, resulting in much higher biofilm pH. This method avoided the low cariogenic pH of commercial orthodontic cements that could lead to WSL [66]. 

Such an ability to raise the pH is important, as Dawes indicated that acidogenic bacteria in biofilms can metabolize carbohydrates to acids and cause a local plaque pH to decrease to 4.5 or even 4 after a sucrose rinse [67]. This can damage the teeth because below pH of about 5.5, tooth demineralization dominates, resulting in a net enamel mineral dissolution [67]. To prevent enamel demineralization around the orthodontic brackets, the local pH needs to be maintained at greater than 5.5. In Figure 4, the two commercial controls with biofilms produced pH below 5. VT had a higher pH than TB control, likely because the fluoride ion release from VT contributed to reducing the acid production of the bacteria. Shinohara et al. showed that fluoride ions could suppress the metabolic pathways such as the fermentation pathway for lactic acid production [68]. Therefore, VT had lower cfu and lower metabolic activity and lactic acid production of biofilms than TB control. However, VT still had biofilm pH in the cariogenic zone. By contrast, the additional protein-repellent ability of VT with 3% MPC was beneficial to further reduce the lactic acid production of bacteria, and effectively raised the pH to a safe zone of around 6.5 to avoid mineral loss. Furthermore, it was shown that low pH 4 in the plaque around orthodontic brackets adversely affected the remineralization process; higher fluoride concentration failed to suppress demineralization at low pH [63,64]. Therefore, a higher pH of above 6 is critically important to tilt the balance toward remineralization. Zhang et al. showed that even in the presence of sucrose with microcosm biofilms, the incorporation of 3% MPC into RMGIs was able to maintain the local pH at a safe level of 6.5 [66]. This had two benefits: It maintained the pH in the safe zone to avoid tooth demineralization; and (2) it enhanced the fluoride remineralization efficacy of RMGIs due to higher pH environment [66]. Further study is needed to investigate the effects of combining MPC, pH increase, and fluoride ions on WSL inhibition in vivo. 

## 5. Combination of Protein-Repellency with Nanoparticles of Silver (NAg) 

**Figure 5:**
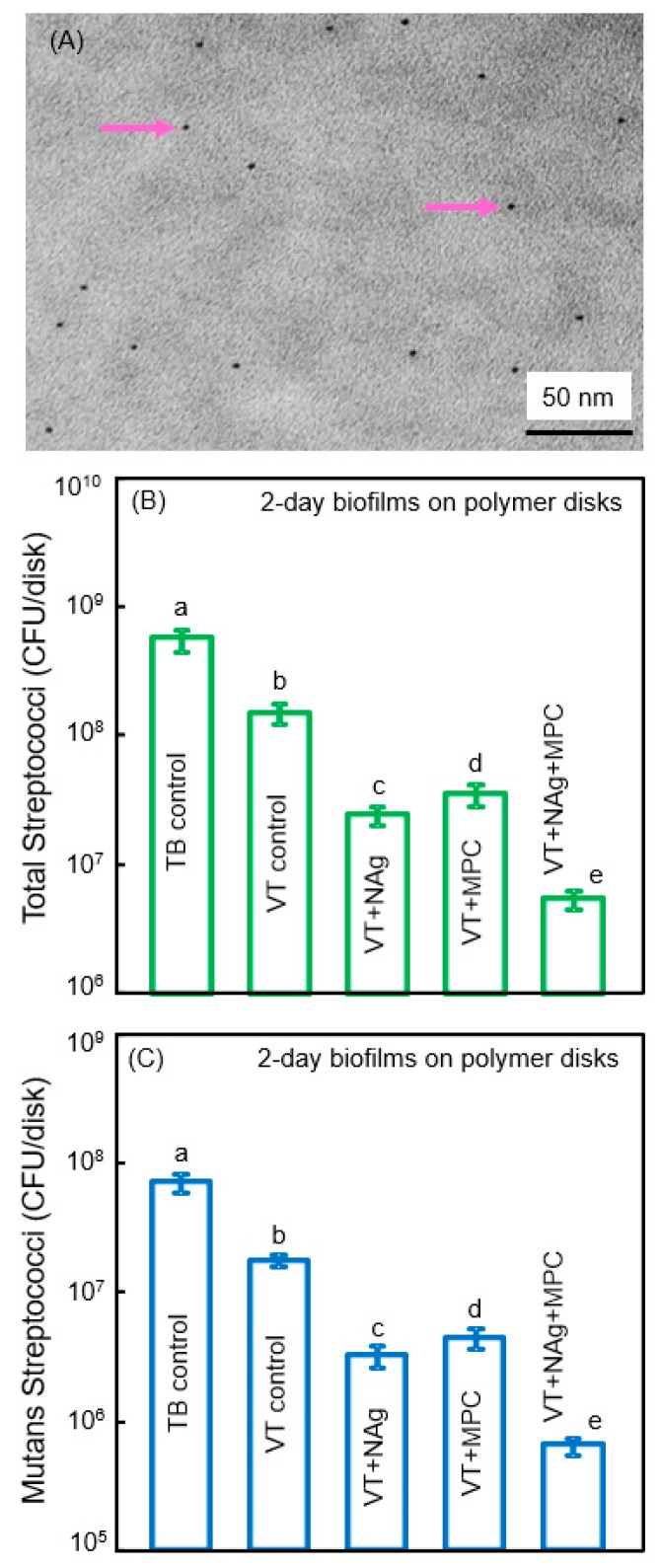
Combining nanoparticles of silver (NAg) with MPC. (**A**) Representative TEM image of NAg (arrows) in resin. The particle size for NAg (mean ± SD; *n* = 100) was (2.7 ± 0.6) nm; (**B**,**C**) Colony-forming units (cfu) of 2-day biofilms on cement with total streptococci and (**C**) mutans streptococci (mean ± SD; *n* = 6). cfu on VT + 0.1% NAg + 3% MPC were 2 logs lower than TB control. In each plot, values with dissimilar letters are significantly different (*p* < 0.05). (Reproduced with permission from [70]. Elsevier, 2015)

Cheng et al. synthesized antibacterial dental polymers containing silver nanoparticles. They used 0.1 g of silver 2-ethylhexanoate (Strem, Newburyport, MA, USA) which were dissolved into 0.9 g of 2-(tert-butylamino)ethyl methacrylate (TBAEMA) [23,69]. TBAEMA was used because it could increase the solubility by forming Ag–N bonds with Ag ions to enhance the Ag salt to dissolve in the monomer solution [23,69]. In addition, TBAEMA contained reactive methacrylate groups which could bond chemically in the polymer matrix. This produced nanoparticles of silver (NAg) that were dispersed in the polymer matrix (Figure 5A). This method yielded NAg with a mean particle size of approximately 2.7 nm [70]. Zhang et al. incorporated NAg into the resin-modified glass ionomer VT at a silver 2-ethylhexanoate/(VT + silver 2-ethylhexanoate) mass fraction of 0.1% [70]. Incorporating 0.1% NAg into VT caused no noticeable change in the color of the paste, compared to VT control. In addition, 3% MPC was also incorporated into VT. The incorporation of 0.1% NAg and 3% MPC into VT did not negatively influence the enamel bond strength, compared to VT control [70]. 

Zhang et al. showed that the incorporation of MPC or NAg each decreased the biofilm cfu, compared to controls (Figure 5B,C) [70]. However, VT + Nag + MPC had a much stronger antibacterial potency than using either MPC or NAg alone. The combined incorporation of MPC and NAg had several merits. First, MPC repelled protein adsorption and bacterial adhesion. Second, incorporation of NAg helped suppress biofilm growth to a level much lower than that achieved via MPC alone. Ag had good biocompatibility and low toxicity to human cells, and induced less bacterial resistance than antibiotics [71]. Regarding the antibacterial mechanism of Ag, studies indicated that the Ag ions could inactivate the vital enzymes of bacteria, rendering the bacterial DNA to lose its replication ability, thus causing cell death [71,72]. Due to the extremely small particle size of 2.7 nm and the high surface area of the nanoparticles, NAg were shown to have strong antibacterial activities [69,70]. Indeed, the NAg addition into dentin bonding agent and orthodontic cement effectively suppressed the oral biofilm growth [23,69]. However, color and esthetics are important for dental applications, which limit the amount of NAg to be incorporated in the polymer. There was no noticeable color change from 0% to 0.1% NAg in the VT, but the color turned darker at 0.15% NAg [70]. Therefore, the optimal NAg concentration in VT appeared to be 0.1%, to obtain a strong antibacterial function without compromising the material’s esthetics. 

The third merit of using NAg in VT for orthodontic applications addressed the clinical problem that the most common sites for demineralization in tooth enamel were around the cements and brackets [61]. This means that it would be desirable for the orthodontic cement to inhibit not only the bacteria on the cement, but also the bacteria in the vicinity away from the brackets, in order to protect the nearby enamel surfaces. Although VT had fluoride release, the antibacterial ability of fluoride was small, its release occurred primarily beneath the brackets, and it was ineffective in preventing demineralization away from the location of the brackets [61,63]. On the other hand, studies indicated that dental polymers containing NAg had a long-distance killing capability, and could kill the bacteria away from the polymer surface, which was achieved by the release of Ag ions [71]. Furthermore, its antibacterial activity was relatively long-term. Yoshida et al. demonstrated that an Ag-containing polymeric composite was able to continue to inhibit *S. mutans* growth when tested for a duration of 6 months [73]. This was consistent with a bonding agent containing NAg which was water-aged for 6 months, and it still possessed an anti-biofilm potency that was similar to that at 1 day [74]. Another potential merit of NAg incorporation into VT was that, while the NAg could inhibit biofilm growth, the fluoride ions from VT could combat demineralization of enamel. These two actions together may be much more effective than a single action to inhibit WSL. Further studies are needed to investigate the release of Ag ions and fluoride ions simultaneously, and to evaluate their possible synergistic effects on caries prevention.

## 6. Tooth Surface Coatings Containing Calcium Phosphate Nanoparticles for Remineralization

Another promising application for nanostructured polymeric materials with protein-repellent and anti-caries properties is to address the prevalence of tooth root caries. The occurrence of root caries increases with aging, which is a growing public health issue due to the rapid growth of the elderly population and the increase in their tooth retention rate [75]. The occurrence of root caries can be increased due to gingival recession in seniors, periodontal disease, or traumatic tooth-brushing actions [75]. Low salivary flow in the elderly and in patients with dry mouths also contributes to the buildup of oral biofilms and plaque [76]. Root caries in the United States increased from 7% among young people to 56% in seniors who are 75 years of age or older [8]. Since the thin cementum coating on tooth roots can be lost due to tooth-brushing or biofilm acid attacks, the root dentin is often exposed after gingival recession [77]. The exposed dentin mineral is known to be more soluble than enamel due its higher carbon content [78]. As a result, demineralization in the tooth roots is twice as fast as that in enamel [79]. Therefore, it would be highly desirable to develop a bioactive and therapeutic coating material to seal and protect the exposed root dentin. 

**Figure 6:**
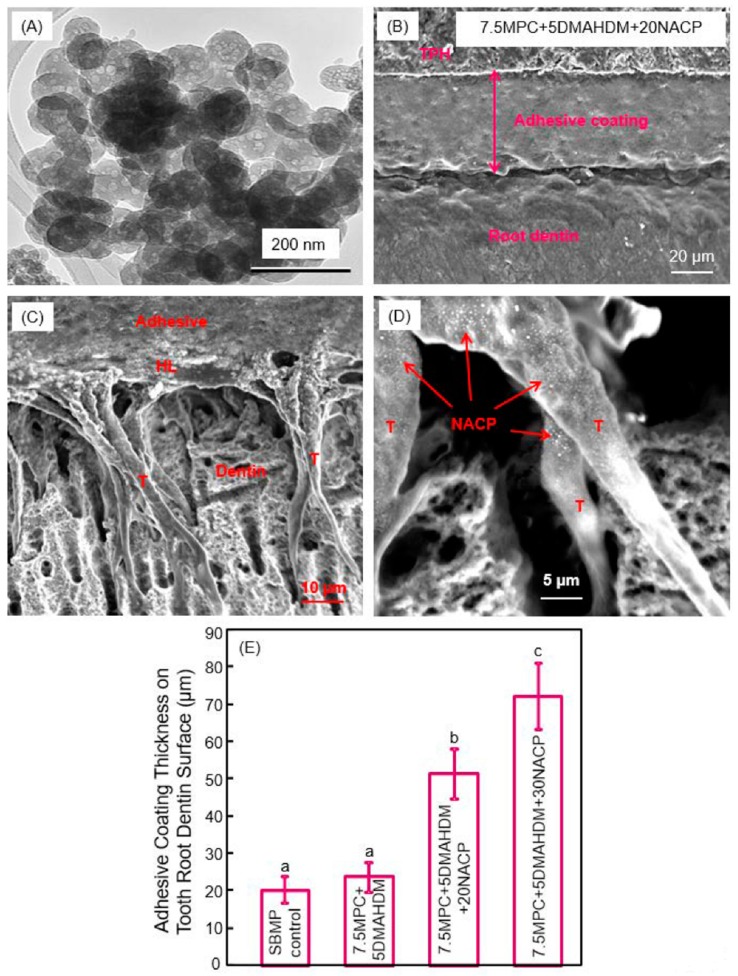
Nanoparticles of amorphous calcium phosphate (NACP) in protein-repellent and antibacterial tooth root coatings. (**A**) SEM image of NACP; (**B**) SEM image of tooth root coating; (**C**) Hybrid layer (HL) and resin tags (T); (**D**) NACP flowed with adhesive into dentinal tubules; (**E**) Incorporation of NACP increased the coating thickness to protect tooth roots (mean ± SD; *n* = 6). Dissimilar letters indicate values that are significantly different from each other (*p* < 0.05). (Reproduced with permission from [90]. Elsevier, 2015)

Calcium phosphate (CaP)-filled dental polymers could release supersaturating levels of calcium (Ca) and phosphate (P) ions to remineralize tooth lesions [80,81,82,83,84]. Nanoparticles of amorphous calcium phosphate (NACP) with particle a size of 116 nm were synthesized via a spray-drying technique, and used as fillers in dental polymers (Figure 6A) [85,86]. NACP nanocomposite achieved Ca and P ion releases similar to those of traditional CaP composites using particles of several microns to tens of microns; however, the nanocomposite possessed much better mechanical properties to support chewing forces orally [85,86]. Due to Ca and P ion release and acid-neutralization capability, the NACP nanocomposite regenerated the lost minerals in the tooth lesions, and inhibited caries at the restoration margins in a human in situ model [87,88,89]. NACP were also incorporated into adhesive cements that could bond to tooth structures. An example of the adhesive coating thickness on the tooth root dentin is shown in Figure 6B [90]. It had a relatively uniform coating, and the exposed dentin was completely sealed by the polymer. Resin tags “T” from the well-filled dentinal tubules were visible in Figure 6C [90]. “HL” refers to the hybrid layer where the cement paste infiltrated the collagen fibers in the dentin to achieve an effective bonding. Arrows in (D) indicate examples of NACP in dentinal tubules, indicating that the NACP were small enough to flow with the cement into the tubules to remineralize the dentin. The coating thicknesses are plotted in Figure 6E [90]. Adding NACP increased the adhesive coating thickness to effectively seal the exposed root dentin, to provide a volume of Ca and P ion reservoir and protect the tooth structures [90].

This bioactive coating cement could have important clinical applications, because the cementum on root surfaces is the first target for biofilms to attack. The natural cementum can be easily removed by root planing during the treatment of periodontal diseases or by excessive tooth-brushing [77]. This, in turn, causes the underlying dentin to be exposed, leading to dentin hypersensitivity and root dentin caries [79]. A bioactive polymer coating on the exposed root dentin could play an important role in protecting the dentin from physical, chemical, and biological stimuli [91]. Therefore, the protective polymer cement containing NACP, MPC, and DMAHDM has great potential to be used to cover the exposed root dentinal surfaces, eliminate dentin hypersensitivity, and inhibit root caries via remineralization ions and protein-repellent and antibacterial functions.

## 7. Therapeutic Restorations to Suppress Periodontal Pathogens 

Therapeutic restorations refer to restorations that not only replace the missing tooth structures, but also exert inhibitory effects against oral diseases, such as the suppression of cariogenic and periodontal pathogens, and exert healing effects, such as releasing agents into the pulp to heal the pulp, or remineralizing and regenerating the lost minerals. As the world population ages, major changes in oral disease patterns occur [92]. For example, there is a significant increasing trend of root caries in senior people. Root caries can be treated with Class V restorations. However, they often have subgingival margins, which are difficult to clean and can provide pockets for periodontal bacterial growth. This, in turn, leads to the worsening of periodontitis and the damage of the periodontal attachment. Oral biofilms are the primary aetiological factor of periodontitis, which can lead to periodontal attachment loss and tooth loss [93]. To make matters worse, the currently available dental polymer-based Class V composites not only have no antibacterial effect, but they actually accumulate more oral biofilms and plaque than other materials, such as metals that are not esthetic [17].

The subgingival plaque of periodontitis and peri-implantitis sites contain bacterial species including *Porphyromonas gingivalis* (*P. gingivalis*), *Prevotella intermedia* (*P. intermedia*), and *Aggregatibacter actinomycetemcomitans* (*A. actinomycetemcomitans*) [94]. Studies have shown that they secrete virulence factors in the periodontal pockets to cause gradual loss of the alveolar bone and periapical bone [94]. Among them, *P. gingivalis* may act as a keystone pathogen in periodontitis [94,95]. It can impair innate immunity in ways that alter biofilm growth and induce a destructive shift in the normally homeostatic host-microbiota interplay in the periodontium [95]. *P. intermedia* is associated with pregnancy gingivitis and periodontitis, as it can use estrogen and progesterone as an essential source of growth [96]. *A. actinomycetemcomitans* has been shown to be associated with localized aggressive periodontitis [97]. In addition, another species, *Fusobacterium nucleatum* (*F. nucleatum*), can co-aggregate with many other plaque bacteria and behave as a microbial bridge between the early and late colonizers [98]. *F. nucleatum* is also an initiator organism that can enhance the physicochemical changes in the gingival sulcus to allow the periodontal pathogenic successors to establish and multiplier [99]. 

**Figure 7:**
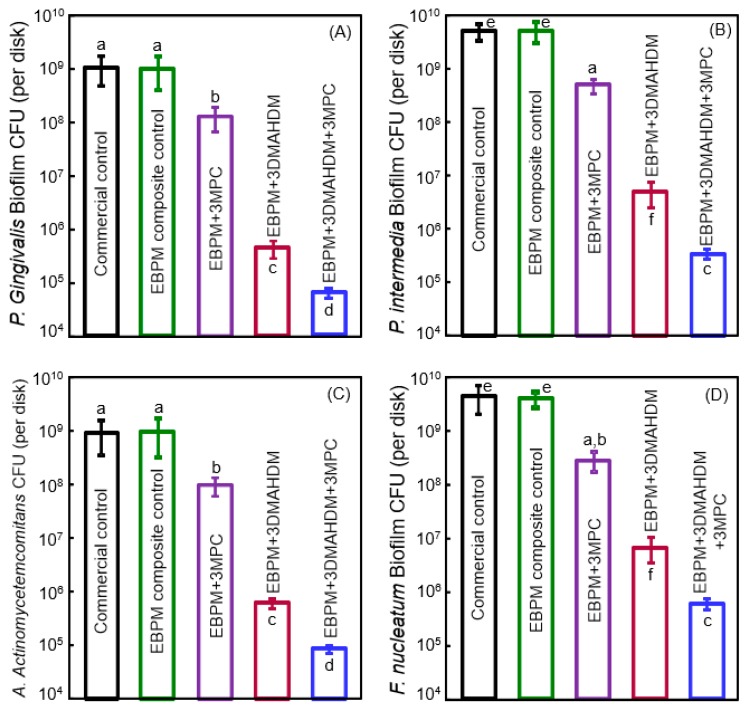
Bioactive Class V composite for tooth root cavities inhibiting four species of periodontal pathogens. cfu of 2-day biofilms on composites: (**A**) *P. gingivalis*; (**B**) *P. intermedia*; (**C**) *A. actinomycetemcomitans*; and (**D**) *F. nucleatum* (mean ± SD; *n* = 6). Note the log scale for the y-axis. Bars with dissimilar letters are significantly different from each other (*p* < 0.05). (Reproduced with permission from [100]. Elsevier, 2016)

To suppress periodontitis-related pathogens, Wang et al. developed a novel therapeutic polymer composite for Class V restorations [100]. The polymer matrix of this composite consisted of ethoxylated bisphenol A dimethacrylate (EBPADMA) and pyromellitic glycerol dimethacrylate (PMGDM) at a mass ratio of 1:1 (referred to as EBPM) [100]. The composite contained 20% NACP for remineralization, 50% glass particles for mechanical strength, 3% MPC for protein-repellency, and 3% DMAHDM for antibacterial function [100]. The mechanical properties showed that adding 3% MPC and 3% DMAHDM did not compromise the strength and elastic modulus, which matched those of a commercial control composite that had no therapeutic effect [100]. Protein adsorption on the composite was decreased by about an order of magnitude via MPC. The cfu counts of 2-day biofilms of periodontal pathogens on this therapeutic composite with MPC and DMAHDM were greatly reduced (Figure 7) [100]. The composite with EBPM + 3% DMAHDM + 3% MPC exerted slightly different inhibition efficacy against the different species, reducing the cfu by slightly less than 4 logs for some species, and more than 4 logs for other species. In general, however, the periodontal pathogen biofilms were reduced by about 4 logs via the therapeutic composite EBPM + 3DMAHDM + 3MPC. Furthermore, the metabolic activity and the polysaccharide production by the periodontal pathogen biofilms were also substantially reduced on the EBPM + 3DMAHDM + 3MPC composite, compared to control composite [100].

Periodontal disease is prevalent worldwide, especially in developing countries. It often leads to tooth loss and decrease in quality of life, is an expensive public health problem [101], and often requires the need for alveolar bone graft, titanium implants, and crowns. Even in developed countries such as the United States, periodontal disease inflicts almost half (45.9%) of the population who are 30 years of age and older [101,102]. Class V restorations with subgingival margins are difficult to clean with pockets for periodontal pathogen growth. This sets off a vicious cycle, causing more gingival recession, which in turn, causes more root exposure and root caries. Therefore, the therapeutic EBPM + 3DMAHDM + 3MPC nanocomposite could be highly beneficial for clinical applications in Class V restorations. Its potent antibacterial function against periodontal pathogens by reducing biofilm cfu by almost 4 orders of magnitude may help inhibit local periodontitis and protect the periodontal attachment. In addition, it contained 20% NACP with Ca and P ions for remineralization [87,88,89] and strengthening of tooth root structures. Further study in this promising direction is needed to realize these potential clinical benefits.

## 8. Long-Term Durability of Bioactive and Therapeutic Dental Polymers

Although these bioactive and therapeutic properties are beneficial, they are required to have long-term durability to be successful clinically. For example, a key requirement for a polymeric adhesive is the long-term endurance of the dentin bond strength. Unfortunately, water adsorption from saliva and drinks is unavoidable in the mouth, especially with the hydroxyl groups in the bonding agents [103]. Water adsorption leads to hydrolysis of the hydrophilic resin [104,105]. Furthermore, at the tooth-restoration margins, the host-derived matrix metalloproteinases (MMPs) have been shown to lead to the dissolution of the exposed collagen fibrils in the hybrid layer [106,107]. The dissolution of collagen may lead to increases in water content, which further degrades the collagen and causes the deterioration of the dentin-polymer bond. In addition, since the degree of polymerization conversion is less than 100%, small amounts of uncured monomers and the breakdown products of the tooth-restoration margins can diffuse out, contributing to the decrease in bond strength. 

**Figure 8:**
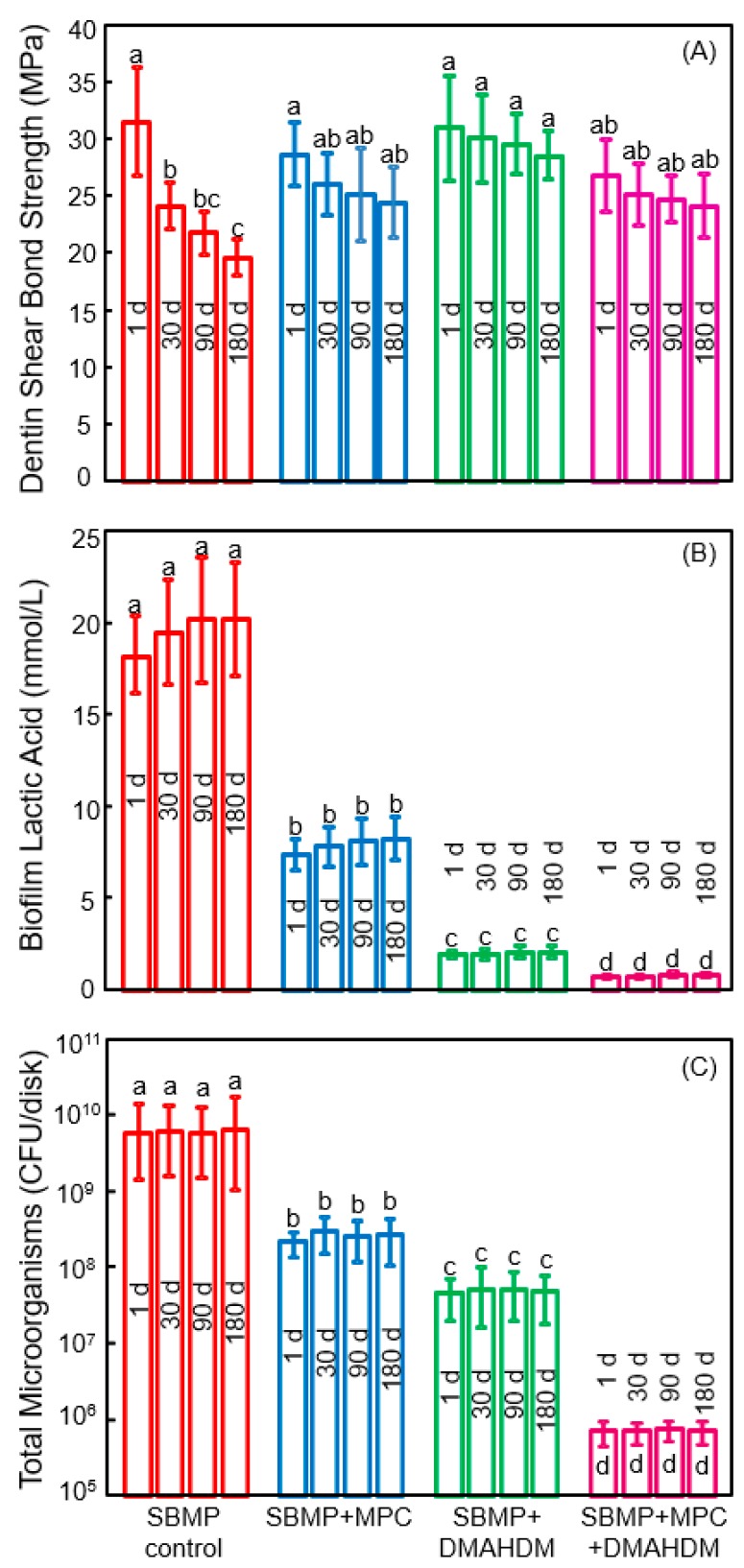
Effects of 6 months of water-aging on long-term durability. (**A**) Dentin bond strength. The bond strength of SBMP decreased during 180 days (*p* < 0.05). There was no significant strength loss for those with MPC and DMAHDM (*p* > 0.1); (**B**) Biofilm lactic acid (mean ± SD; *n* = 6); (**C**) Colony-forming units (cfu) for total microorganisms (mean ± SD; *n* = 6). cfu on SBMP+MPC+DMAHDM was nearly 4 logs less than that of SBMP control (*p* < 0.05). For each group, there was no significant difference in cfu before and after 6 months of water-aging (*p* > 0.1). In each plot, values with dissimilar letters are significantly different (*p* < 0.05). (Reproduced with permission from [108]. Spinger Nature, 2018)

The new generation of therapeutic bonding agents with protein-repellent and antibacterial functions are promising to enhance the longevity of the dentin-polymer bond strength. In a recent study, Zhang et al. investigated the durability of the protein-repellent adhesives incorporating MPC and DMAHDM (Figure 8) [108]. The dentin shear bond strengths vs water-immersion time from 1 day to 180 days are plotted in Figure 8A. The bond strength of the commercial SBMP control significantly dropped during 180 days of water-immersion. For the groups with MPC and DMAHDM, although there was a slight decrease in bond strength with increasing time, the decreases were not significant (*p* > 0.1). At 180 days, SBMP + MPC, SBMP + DMAHDM, and SBMP + MPC + DMAHDM all had significantly greater dentin bond strength than SBMP control (*p* < 0.05). The groups containing MPC had protein amounts that were about 1/20 that of SBMP control. Water-aging the polymers for 180 days prior to the protein adsorption test had no effect on protein amounts, demonstrating that the protein-repellency did not decline with increasing water-aging time [108]. Water-aging the polymers from 1 to 180 days did not affect the biofilm acid production (Figure 8B). DMAHDM + MPC + DMAHDM had the least lactic acid from oral biofilms, which was nearly 1/20 that of SBMP control. The total microorganism cfu counts of two-day oral biofilms on the polymers are plotted in Figure 8C. For each group, there was no difference in cfu with water-immersion from 1 to 180 days (*p* > 0.1). Adding MPC or DMAHDM each alone into the polymer reduced the biofilm cfu, compared to SBMP control (*p* < 0.05). In sharp contrast, using the MPC + DMAHDM combination, the SBMP + MPC + DMAHDM had much lower cfu than those using MPC or DMAHDM alone. The biofilm cfu on SBMP + MPC + DMAHDM was nearly 4 logs less than SBMP control, even after 180 days of water-aging, demonstrating the long-term and durable anti-biofilm function [108]. 

The reason that the bioactive groups had greater dentin bond strengths at 180 days than SBMP control was likely because that QAMs had MMP-inhibitory and anti-enzyme activities [109]. The dentin bond strength of SBMP decreased after 180 days of water-aging, which is typical for commercial bonding agents. By contrast, Zhang et al. demonstrated that the novel bioactive bonding agents with DMAHDM and MPC exhibited no significant decrease in bond strength from 1 to 180 days [108]. DMAHDM was copolymerized in the polymer matrix and was not leached out or lost over time, therefore, it provided long-lasting effects. MPC was also copolymerized in the polymer matrix for durable effects. MPC may also have anti-MMP activity because MPC contains a quaternary ammonium group [110], which is analogous to that in QAMs. Furthermore, MPC contains a negatively-charged phosphate group [110], which may allow MPC to exert electrostatic interactions and influence the configuration of the active sites of MMPs, thereby exerting an inhibitory effect on MMPs [109]. Future studies are needed to further elucidate the underlying mechanisms via which the DMAHDM + MPC adhesive maintained its dentin bond strength without degradation. The novel adhesive with MPC and DMAHDM achieved long-term dentin bond strength significantly beyond current commercial materials, plus durable resistance to proteins and oral bacteria. Therefore, this bioactive and therapeutic polymer is promising for dental applications to reduce biofilm formation, suppress caries, and increase the longevity of the restorations.

## 9. Conclusions

This article reviewed current research efforts in developing a new generation of dental restorations with protein-repellent, anti-biofilm, and anti-caries capabilities. Unlike traditional materials which are generally bio-inert, the new generation is bioactive and therapeutic, with capabilities to repel proteins, inhibit pathogens, reduce or eliminate biofilm acids, raise biofilm pH and regenerate lost tooth minerals. This new generation employs agents including QAMs, protein-repellent agent, silver nanoparticles, and calcium phosphate nanoparticles, with applications in dental polymer composites, bonding agents, cements and coatings. They can be combined with fluoride release and reinforcement fillers for optimal properties. They have been shown to be highly effective against not only cariogenic biofilms, but also periodontal pathogens. Furthermore, their bioactive and therapeutic effects have been demonstrated to be durable and long-lasting. This new generation of dental biomaterials offers the much-needed healing, therapy, and regeneration capabilities that are lacking in traditional materials, and hence, is promising in terms of improving a wide range of dental treatments.
